# Exploring Mortality and Associated Risks Among Assisted Home Hemodialysis Patients in Qatar

**DOI:** 10.1111/hdi.13236

**Published:** 2025-03-28

**Authors:** Abdullah I. Hamad, Amani Z. Zidan, Mostafa F. Elshirbeny, Fadwa S. Al‐Ali, Tarek A. Ghonimi, Mohamed Y. Abdelhadi, Mossab Filali, Ahmed Awaisu, Rania A. Ibrahim, Mohamad M. Alkadi, Hassan A. Al‐Malki

**Affiliations:** ^1^ Division of Nephrology, Department of Medicine Hamad Medical Corporation Doha Qatar; ^2^ Department of Clinical Pharmacy and Practice College of Pharmacy, QU Health, Qatar University Doha Qatar; ^3^ Metcocare Company Doha Qatar

**Keywords:** all‐cause mortality, assisted home Hemodialysis, chronic kidney disease, end‐stage kidney disease, hemodialysis, mortality risk factors

## Abstract

**Background:**

Home hemodialysis (HD) is a well‐established modality that promotes patient independence but poses significant challenges, particularly in regions like the Gulf Cooperation Council (GCC) countries. Assisted home HD has gained momentum in the GCC over the past few years. Qatar's assisted home HD program has expanded substantially in the past 2 years. This study examines the demographics, mortality rates, and associated risk factors within Qatar's assisted home HD patient population.

**Methods:**

This was a retrospective cohort study to review national data for all assisted home HD patients in Qatar between July 2021 and December 2023. Patients had to be > 60 years old and have limited mobility to be eligible for the assisted home HD program and included in the study.

**Results:**

We had 114 assisted home HD patients with a median age of 71.5; 54 (47.4%) were males. During the study period, 20 patients (17.5%) died, and 8 (7%) stopped receiving the service for traveling abroad, personal preference for in‐center HD, or changing dialysis modality. Most deaths occurred due to infectious causes. The deceased patients had significantly less HD vintage, more severe immobility, and more hospitalizations compared to the alive participants. On multivariate analysis, patients with severe immobility had 3.8 (CI: 1.1–12.8, 95% *p* < 0.05) times higher odds of mortality than patients with mild to moderate immobility.

**Conclusion:**

Our study found that mortality in the assisted home HD program is significant and mostly related to mobility status. Patients with severely reduced mobility had almost four times the risk of mortality compared to more mobile patients. Further, larger studies are needed to confirm these findings.

## 1 Background

Home hemodialysis (HD) is a type of renal replacement therapy typically performed by patients or their caregivers at their homes. Alongside peritoneal dialysis, home HD is classified as a home dialysis modality. After undergoing standard training, patients are equipped to carry out the therapy independently, with the support of remote monitoring and periodic visits from the medical team [1]. These home‐based dialysis therapies provide patients with greater freedom, enhanced self‐management, and an improved quality of life [2, 3]. Traditional home HD has faced significant barriers to widespread adoption in the Gulf Cooperation Council (GCC) region due to a variety of challenges, including cultural factors, logistical constraints, and patient acceptance [4]. As a result, it has not gained the same traction in the GCC as in other regions. In response to these challenges, assisted home HD has emerged as a modified version of home HD. In assisted home HD, dialysis treatments are typically performed by a trained visiting hemodialysis nurse, with the full and direct supervision of the medical team. This approach reduces the burden on patients, as they have minimal responsibilities in terms of performing the dialysis procedure on their own.

Assisted home HD remains a niche modality, with very few programs implemented globally and limited published research in this area. Notably, Agraharkar et al. documented a restricted experience with assisted home HD in terminally ill patients, highlighting its potential application in this unique population [5, 6]. Similarly, Abdelwahab et al. and Berniah et al. provided insights into assisted home HD outcomes within a cohort of homebound, debilitated patients in the United Arab Emirates, reporting their findings in 2019 and 2020, respectively [7, 8]. Over the past two decades, the dialysis population in Qatar has experienced steady growth, reflecting the increasing prevalence of end‐stage kidney disease (ESKD) in the region [9]. Notably, the home dialysis population, encompassing both peritoneal dialysis (PD) and assisted home HD, has expanded significantly, particularly during the COVID‐19 pandemic [10, 11]. Currently, home‐based therapies account for approximately 20% of Qatar's total dialysis population, underscoring the country's commitment to diversifying dialysis modalities and addressing the unique needs of its patients [12, 13]. In our publication by Hamad et al., we detailed the successful establishment of Qatar's assisted home HD program, including the steps taken and challenges encountered during its initiation [13]. The program utilizes traditional HD machines rather than portable units, with a dedicated dialysis setup installed in a designated room within the patient's home. Specialized training was provided to assisted home HD staff focused on the home dialysis setting, including effective social interaction with patients and their families, managing complications unique to home dialysis, adherence to clinical protocols and policies, and making informed decisions in diverse home environments. Our assisted home HD program is primarily designed to cater to elderly HD patients, particularly those with limited mobility. Most of these patients rely on ambulance services for transportation to and from dialysis centers, highlighting the logistical and physical challenges they face [13].

Traditional assisted home HD has been associated with favorable outcomes, with literature suggesting mortality benefits for short, frequent assisted home HD treatments compared to in‐center hemodialysis [14, 15, 16, 17]. However, there is a notable gap in research regarding assisted home HD, particularly concerning mortality outcomes.

In this study, we aimed to address this gap by examining the demographics and identifying key risk factors predicting mortality within our assisted home HD population.

## 2 Study Objectives

This study primarily aimed to analyze all‐cause mortality among patients undergoing assisted home HD in Qatar and to identify key contributing factors. Secondarily, it focused on examining patient characteristics, demographics, and comorbidities.

## 3 Materials and Methods

### 3.1 Study Design

This retrospective cohort study included chart reviews for all chronic hemodialysis patients in Qatar. We screened all patients receiving their dialysis at Hamad Medical Corporation, the sole provider of HD services in Qatar, between July 2021 and December 2023 and included those eligible for assisted home HD service.

### 3.2 Institutional Review Board

The study was conducted in accordance with the Declaration of Helsinki and approved by the Institutional Review Board for the Medical Research Center at Hamad Medical Corporation Qatar (MRC‐01‐23‐781; February 20, 2023 and MRC‐01‐24‐014; June 13, 2024).

The IRB waived the informed consent, given the study's retrospective design.

### 3.3 Study Population

**FIGURE 1:**
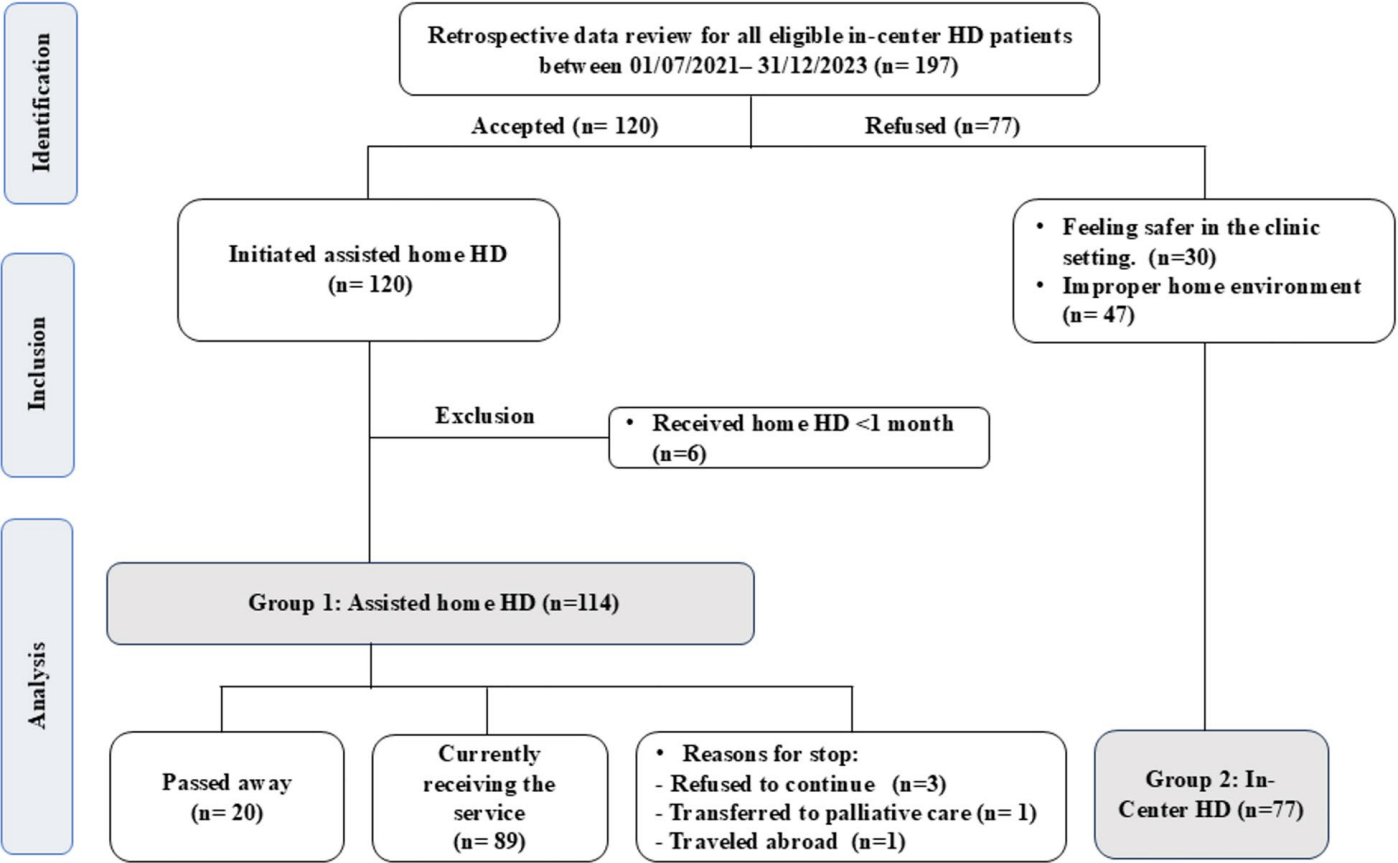
Flow diagram for all ambulatory hemodialysis patients in Qatar who are receiving their dialysis in Hamad General Hospital and are eligible for assisted home hemodialysis (HD) service between July 1, 2021 and 2031 (December 2023).

Based on the medical records review, 197 adult HD patients eligible for the assisted home HD service were screened between July 2021 and December 2023. Among them, 120 cases shifted from in‐center HD to assisted home HD service (Figure 1).

#### 3.3.1 Inclusion Criteria for the Assisted Home HD Study

- Age older than 60 years.
- On HD for at least 3 months.
- On assisted home HD ≥ 1 month.
- Have functional HD access.
- Maintain a suitable home environment, including a private room with consistent water and electricity supplies.
- Limited mobility as the following:
  ○ **Severe immobility:** bedridden and using an ambulance for transportation to the dialysis unit.
  ○ **Mild to moderate immobility:** wheelchair bound, using walking aid or assistance.

#### 3.3.2 Exclusion Criteria

- Acute kidney injury.
- HD < 3 months.
- Peritoneal dialysis.
- Ambulatory.
- Assisted home HD < 1 month.

#### 3.3.3 Study Groups

1. **Home HD:** Eligible patients who met the inclusion criteria and received the service during the studied period.
2. **In‐center HD:** Eligible patients who met the inclusion criteria but preferred the in‐center HD.

### 3.4 Study Aims

1. Assessing primary objective: Mortality was calculated as all‐cause mortality among patients eligible for home HD during the study duration.
2. For secondary Objective: Patient characteristics, demographics, comorbidities, and mobility status were assessed for potential risk factors for mortality.

### 3.5 Program Details

Our assisted home HD program is conducted in a dedicated room within the patient's home. The room is equipped with a standard hemodialysis machine (Nikkiso DBB EXA/DBB EXA ES) and a portable reverse osmosis system. Additionally, all necessary water connections, including plumbing, drainage, a water tank, and a water chiller, are installed to ensure seamless operation. Dialysis sessions are performed three times per week, each lasting 4 h, following the same protocol as our in‐unit dialysis treatments. The program adheres to the same guidelines and performance measures as our dialysis centers, ensuring optimal management of anemia, mineral bone disorders, treatment adequacy, and so forth.

### 3.6 Data Collection

All cases were identified from Qatar's national electronic medical records at HMC (CERNER Millennium Software is an electronic health record system used in Hamad Medical Corporation to manage patient information and clinical workflows) to retrieve the following data: demographic data (age, gender, dialysis treatment duration), comorbidities, dialysis adequacy, vascular access types (arteriovenous fistula, arteriovenous graft, permanent catheter), vascular access complications (catheter malfunction, infection, bleeding), hospitalization, incidence of falls, technical incidents episodes, and need for transportation.

### 3.7 Statistical Analysis

Data were analyzed using the statistical package SPSS version 29.0. Data were descriptively summarized using means and standard deviations (normally distributed), medians, and interquartile ranges. Pearson's Chi‐square statistics were used to measure relationships between categorical variables and *t*‐tests or Mann–Whitney U tests for continuous variables. Logistic regression was used to explore the factors associated with mortality. Survival was investigated using Kaplan–Meier survival curves. All tests were two‐tailed, and *p*‐values < 0.05 were considered statistically significant.

## 4 Results

We reviewed the records of all patients eligible for the assisted home HD service in Qatar between July 2021 and December 2023 (*n* = 197). Among these, 30 patients opted to continue with regular in‐center HD due to personal preference, and 47 patients were found to have home environments unsuitable to safely support assisted home HD. The remaining 120 patients successfully started the service. However, we excluded six patients from the analysis as they had undergone less than 1 month of assisted home HD, which was considered insufficient to yield meaningful data for the study (Figure 1).

### 4.1 Patients' Demographics and Comorbidities

The records of 114 patients receiving assisted home HD at the end of 2023 were analyzed and compared with the data of the 77 patients who continued to receive the in‐center HD. The median (IQR) age of the two groups was 71.5 (65–80) and 69 (63.5–79.5) years, respectively. Detailed patient characteristics, clinical data, and technical aspects of HD are summarized for both groups in Table 1. The two groups exhibited a high prevalence of diabetes mellitus (86.8% of the home HD group and 80.5% of the in‐center HD group) and hypertension (95.6% of the home HD group and 96.1% of the in‐center HD group). The median (IQR) of the HD vintage was 38 (19–67) for the home HD and 62 (22–94) for the in‐center HD patients. Mobility status was nearly evenly split, with almost 52% experiencing severe immobility (e.g., bedridden, requiring stretcher assistance, and requiring ambulance transport to the dialysis center) and 48% having mild to moderate limited mobility (e.g., reliance on walking aids, occasional wheelchair use, or restricted mobility range). Additionally, a significant proportion of patients (66.7% in the home HD group, and 66.2% in the other group) utilized a permanent dialysis catheter as their vascular access.

**TABLE 1:** Demographic, clinical, and assisted home HD technical characteristics of the included participants (*n* = 191).

Variables	Home HD group *N* = 114 (%)	In‐center HD group *N* = 77 (%)	*p*
**Age** ^a^	71.5 (65–80)	69 (63.5–79.5)	0.35
**Gender**			
Male	54 (47.4)	25 (32.5)	0.04
Female	60 (52.5)	52 (67.5)	
**Co‐morbidity**			
DM	99 (86.8)	62 (80.5)	0.32
HTN	109 (95.6)	74 (96.1)	0.86
CHF	29 (25.4)	14 (18.2)	0.23
CVA	30 (26.3)	16 (20.7)	0.38
CAD	45 (39.5)	22 (28.6)	0.12
**HD vintage** ^a^	38 (19–67)	62 (22–94)	0.05
**Home HD vintage** ^a^	10 (4–14)	—	—
Total number of hospitalizations^b^	1.1 ± 1.5	2.4 (2.9)	< 0.001
**Mobility**			
Severely impaired mobility	59 (51.8)	40 (51.9)	0.98
Mild to moderate impaired mobility	55 (48.2)	37 (48.1)	
**Vascular access**			0.22
AVF	29 (25.4)	25 (32.5)	
AVG	4 (3.5)	0	
Central Catheter	76 (66.7)	51 (66.2)	
AVF+ Central Catheter	4 (3.5)	1 (1.3)	

Abbreviations: AVF: arteriovenous fistula; AVG: arteriovenous graft; CAD: coronary artery disease; CHF: congestive heart failure; CVA: cardiovascular accident; DM: diabetes mellitus; HD: hemodialysis; HHD: home hemodialysis; HTN: hypertension.

^a^ Median (IQR).

^b^ Mean ± SD, (IQR).

### 4.2 Primary Outcome

Until the conclusion of data collection, patient death, or withdrawal from the service, patients received assisted home HD for a median duration of 10 [4, 5, 6, 7, 8, 9, 10, 11, 12, 13, 14] months. During this period, 20 patients (17.5%) passed away, 1 (3.5%) transitioned to palliative care, 3 (25%) discontinued assisted home HD due to social constraints, and 4 (3.5%) traveled abroad. The causes of death are outlined in Table 2, with sepsis and other infectious complications being the most frequent, accounting for more than half of all deaths. Other causes were varied, including a few cases where the exact cause of death was unclear (*n* = 4).

**TABLE 2:** All‐cause mortality among the study groups (*n* = 191).

Causes	Home HD (*N* = 114)	In‐center HD (*N* = 77)	Total (*N* = 191)
Infections^a^	10 (50)	13 (68.4)	23 (59)
Cardiac‐related causes	4 (20)	3 (15.7)	7 (17.9)
Cerebrovascular accident	1 (5)	1 (5.3)	2 (5.1)
Gastrointestinal bleeding	1 (5)	1 (5.3)	2 (5.1)
Malignancies	0	1 (5.3)	1 (2.6)
Other causes (died at home, died abroad)	4 (20)	0	4 (10.3)
Total	20 (17.5)	19 (24.7)	39 (20.4)

^a^ Sepsis and other infectious complications.

Among the patients who received the assisted home HD service, the differences between deceased and surviving patients were analyzed. Deceased patients generally had a shorter hemodialysis vintage compared to surviving patients (40 [20.74–74.25] months vs. 25 [12–46.75] months; *p* < 0.05). They also exhibited a significantly higher rate of severe immobility requiring ambulance transfers (80% vs. 45.8%; *p* < 0.05) (Table 3). Comorbidities, total number of hospitalizations, and the prevalence of vascular access types were similar between the two groups. Baseline laboratory tests were broadly comparable, with only minor differences observed.

**TABLE 3:** The differences between the variables of expired and alive participants who received the home hemodialysis.

Variables	Expired (*n* = 20)	Alive (*n* = 94)	*p*
**Age** ^a^	74.5 (67–84)	71 (65–79)	0.167
**Gender** ^b^			
Male	6 (30)	48 (51.1)	0.087
Female	14 (70)	46 (48.9)	
**HD vintage**	25 (12–46.75)	40 (20.74–74.25)	0.038
**HHD vintage**	5.5 (3.25–13.25)	11 (5.75–16)	0.073
**Mobility** ^b^			
Severe immobility	16 (80)	43 (45.8)	0.005
Mild to moderate immobility	4 (20)	51 (54.3)	
**Vascular access** ^b^			
AVF	3 (15)	26 (27.7)	
AVG	0	4 (4.3)	0.457
Central Catheter	16 (80)	61 (64.9)	
AVF+ Central Catheter	1 (5)	3 (3.2)	
**Co‐morbidities** ^b^			
DM	19 (95)	80 (85.1)	0.235
HTN	18 (90)	91 (96.8)	0.117
CHF	6 (30)	23 (24.5)	0.606
CVA	4 (20)	26 (27.7)	0.480
CAD	9 (45)	36 (38.3)	0.578
Arrhythmia	8 (40)	26 (27.7)	0.273
**Laboratory values** ^a^			
Baseline HB	11.5 (9.7–12.1)	11.2 (10.1–11.9)	0.803
Bassline iron saturation	27 (20–28.8)	23.5 (19.7–30)	0.771
Baseline ferritin	605 (404.5–1023.3)	528 (306–800)	0.482
Baseline K	5 (4.3–5.3)	5 (4.3–5.5)	0.591
Baseline calcium	2.4 (2.2–2.6)	2.4 (2.3–2.5)	0.445
Baseline phosphorus	1.4 (1.1–1.7)	1.4 (1–1.9)	0.870
Baseline PTH	226 (155–370)	257 (143–411)	0.519
Albumin g/L	33 (29.3–36.8)	35 (33–37)	0.077
Adequacy (URR)	68.9 (62.9–76)	71.9 (66.4–77.1)	0.288
**Total number of hospitalizations** ^c^	1.65 ± 2.18	0.98 ± 1.29	0.198
**Number of technical malfunctions** ^a^	1 (1–1.5)	1 (1–2)	0.498

Abbreviations: AVF: arteriovenous fistula; AVG: arteriovenous graft; CAD: coronary artery disease; CHF: congestive heart failure; CVA: cardiovascular accident; DM: diabetes mellitus; HD: hemodialysis; HHD: home hemodialysis; HTN: hypertension.

^a^ Data are reported as median (IQR).

^b^ Data are reported as *N* (%).

^c^ Data are reported as Mean ± SD.

The overall mortality rate during the study period was higher among the patients who preferred the in‐center HD (*n* = 77) than among the patients who received the home HD (24.7% and 17.5%, respectively).

**FIGURE 2:**
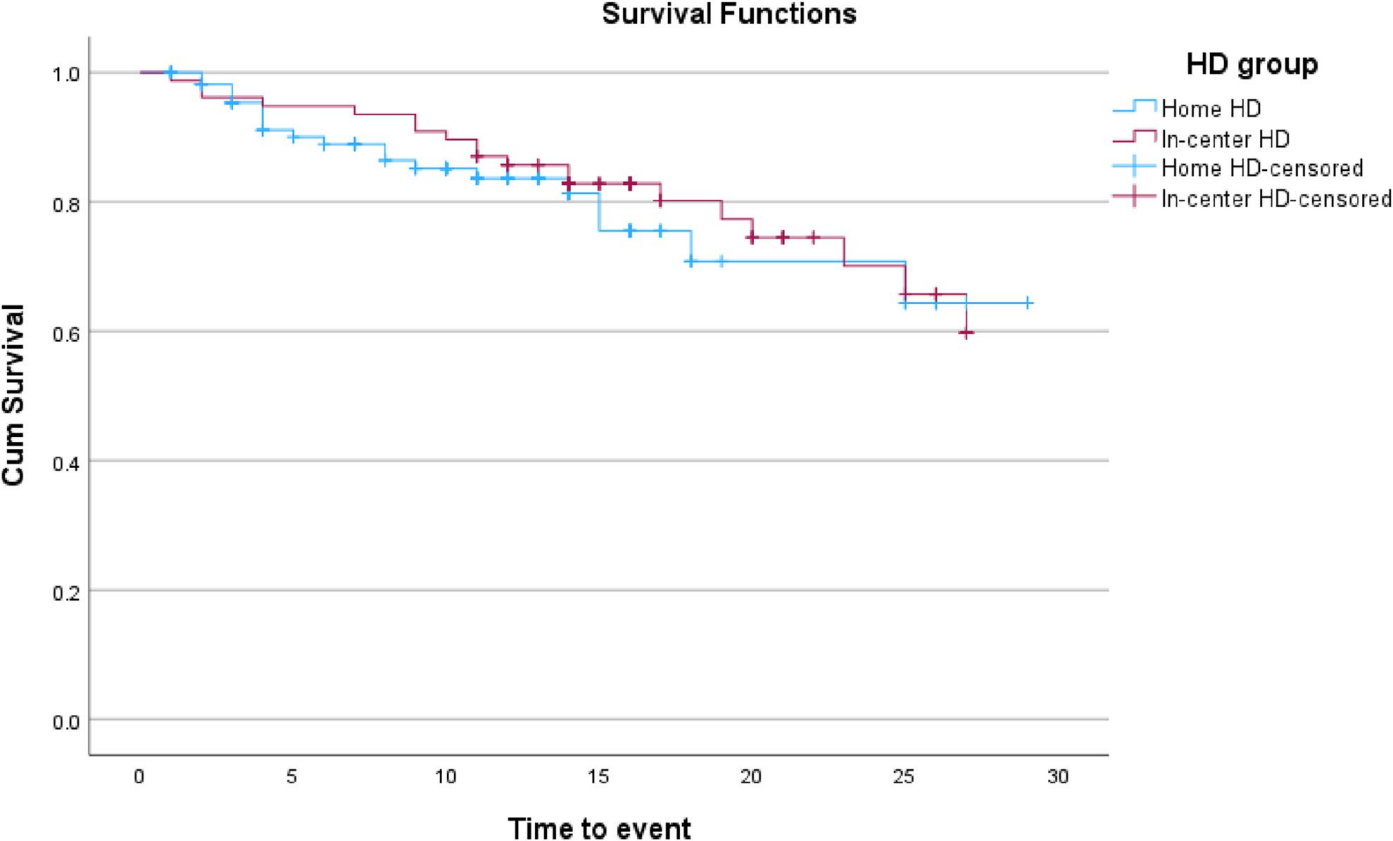
Kaplan–Meier survival plot.

A log‐rank test was run to determine if there were differences in the survival distribution for the two dialysis modalities (HD and home HD). The survival distributions for the two dialysis modalities were not statistically different, χ^2^(2) = 0.24, *p* = 0.62 (Figure 2).

### 4.3 Secondary Outcome: Identifying Risk Factors Associated With Mortality

A binomial logistic regression analysis was conducted to evaluate the effects of age, gender, mobility, hospitalizations, and HD vintage on the likelihood of mortality. Among the five predictor variables, only mobility and HD vintage were found to be statistically significant (Table 4). Patients with severe immobility had a 3.8 times higher risk of mortality compared to those with mild to moderate immobility (95% CI: 1.1–12.8; *p* < 0.05).

**TABLE 4:** Logistic regression predicting the likelihood of mortality based on age, gender, mobility, and number of hospitalizations (*n* = 114).

	*p*	Odds ratio	95% CI	
			Lower	Upper
Age	0.263	1.026	0.981	1.073
Gender	0.155	2.264	0.735	6.973
HD Vintage	0.041	0.980	0.962	0.999
Immobility	0.031	3.82	1.13	12.8
Hospitalization	0.249	1.996	0.617	6.457

## 5 Discussion

Our study investigated mortality and its associated risk factors within Qatar's assisted home HD program. We reviewed the characteristics of 114 patients with end‐stage kidney disease (ESKD) maintained on assisted home HD. We evaluated the all‐cause mortality rate and its contributing factors. A composite outcome of mortality and assisted home HD discontinuation rate was observed in one‐fourth of the patients during the study duration of 30 months (July 2021 to December 2023). This included 20 deaths (17.5% of cases) and eight patients (7%) who discontinued assisted home HD due to reasons such as refusing to continue the assisted home HD program, traveling abroad with loss to follow‐up, or transitioning to palliative care.

These findings align with previous studies, which reported assisted home HD continuation rates of 77%–85% 3 years after initiation [18, 19, 20].

In recent years, the epidemiology of assisted home HD has shifted dramatically, with an increasing number of elderly, diabetic patients and those with multiple comorbidities opting for this modality [21]. Assisted home HD has been consistently associated with better survival rates compared to in‐center HD [20]. For instance, in 2024, Koji et al. reported a 100% survival rate among self‐cannulated home HD patients 2 years after initiation and a 97% survival rate at 3 years [22]. This improved survival is largely attributed to the more frequent use of intensive dialysis compared to in‐center modalities [23]. Additionally, growing expertise in managing home HD patients, particularly in large dialysis centers, has contributed significantly to enhanced patient and technique survival [24]. Home HD patients have also been shown to experience a lower risk of stroke, acute coronary syndrome, and cardiovascular mortality compared to patients on other dialysis modalities [25, 26]. Another critical factor in improving home HD survival is the reduction in infection‐related hospitalizations, particularly in the years following the initiation of home dialysis [27]. This advantage became especially apparent during the COVID‐19 pandemic when home HD patients demonstrated lower rates of COVID‐19 infection compared to their in‐center counterparts [28].

Published literature on assisted home HD is limited and presents varying conclusions due to the unique selection process for patients [5, 6, 7, 8]. Agraharkar et al. reported their experience with assisted home hemodialysis in 28 severely debilitated, terminally ill patients, 82% of whom passed away after a mean follow‐up of 14 months. They concluded that the model was both convenient and comfortable.

Similarly, Abdelwahab et al. and Berniah et al. documented their experiences in the United Arab Emirates within the Gulf Cooperation Council. Their programs included elderly, frail patients who were bedridden at home or in nursing facilities. They utilized portable dialysis machines for 46 and 96 patients, respectively, with a mean patient age of 70 years. The use of tunneled catheters ranged from 46% to 57%. Both studies reported improved quality of life measures, with an annual mortality rate between 11.5% and 17.7%.

Our study aligns with these previous reports in terms of patient selection, age, permanent dialysis catheter use, and immobility. The mortality rate in our cohort was similar between assisted home HD and the comparable population in‐center HD.

Physical mobility impairment is a prevalent issue among dialysis patients, affecting up to 20% of this population [29]. The prevalence is even higher among elderly patients; studies indicate that 57% of elderly hemodialysis patients experience some degree of mobility limitation [30]. This impairment is strongly associated with increased rates of adverse outcomes in dialysis patients [31, 32]. For instance, Garcia et al. reported a significant disparity in emergency service visits between frail and non‐frail hemodialysis patients, with rates of 3216 and 1545 visits per 1000 patient‐years, respectively (*p* < 0.05) [33]. Similarly, mobility limitations significantly influence mortality rates. One study found that the mortality rate was 44% among hemodialysis patients unable to walk, compared to just 9% in those who walked at a speed of ≥ 0.6 m/s [34]. Our findings align closely with previous studies, as impaired mobility emerged as a significant risk factor for mortality in our patient population. Patients with severe immobility had 3.8 times higher odds of mortality (95% CI: 1.1–12.8, *p* = 0.031) compared to those with mild to moderate immobility.

A recent retrospective cohort study evaluating the mortality rate and its causes among chronic ambulatory dialysis patients in Qatar reported an overall crude mortality rate of 6.4%. The study identified age, female gender, and hemodialysis (compared to peritoneal dialysis) as significant risk factors for mortality in this patient population [35]. Additionally, racial differences appear to influence the risk of technique failure and mortality [36, 37]. In the United States, Black patients were the only ethnic group observed to have a higher risk of technique failure compared to White patients, yet they exhibited lower mortality rates [37]. Conversely, a study conducted in Canada found no significant association between race and either technique failure or mortality among home HD patients [38]. In our study, we did not notice any significant association of age, gender, or comorbidities, including diabetes and cardiovascular diseases with mortality. Additionally, we did not find a significant impact of vascular access type on patient survival. Although hemodialysis vintage was statistically significant with *p* < 0.05, odd ratio was 0.98 which meant a reduced risk of death by only 2%.

Although cardiovascular diseases are traditionally the most common cause of death among all CKD patients, including those on hemodialysis [39], the risk of death due to infection is also notably high [40]. In a previous study, among cohort of in‐center hemodialysis patients, cardiovascular causes were the leading contributor to mortality, accounting for 38% of deaths, followed by sepsis, which accounted for 27%. The least common cause of mortality was cerebrovascular disease [35]. The long‐term survival data for assisted home HD show similar mortality causes to those observed in the in‐center dialysis population. A recent study on long‐term home HD survival found that cardiac death was responsible for 50% of fatalities, followed by cancer (25%) and sepsis (17%) [22]. In contrast, in our assisted home HD (and comparable in‐center HD population), infectious diseases were the leading cause of death, accounting for 50% of cases. This could be related to the high rate of permanent dialysis catheters used in our cohort. Cardiovascular events were the second most common cause, contributing to 20% of deaths, followed by cerebrovascular diseases, gastrointestinal bleeding, and other causes.

These findings highlight the need for further studies with larger patient populations and adjustments for comorbidities. Such research would help better understand the causes of mortality and technique failure, ultimately informing the optimal design of home hemodialysis programs.

Our study has several limitations that should be considered when interpreting the findings. First, the relatively small sample size may reduce the statistical power and limit the ability to perform detailed subgroup analyses or draw definitive conclusions regarding patient and technique survival. Second, as the study exclusively included participants from Middle Eastern populations, the findings may lack generalizability to other ethnic groups, preventing the evaluation of long‐term outcomes across diverse racial and ethnic populations. Third, the assisted home HD model has been successfully implemented in the GCC region. However, logistical, financial, and other factors may hinder its adoption elsewhere, limiting its generalizability. Despite these limitations, our study has several notable strengths. First, it contributes to the limited body of research specifically focused on assisted home, distinguishing it as a unique modality compared to conventional home hemodialysis with self‐cannulation. Second, the use of a unified electronic medical record system across in‐center dialysis units, home dialysis units, and hospitals enhances the accuracy and reliability of data collection, particularly for hospitalizations and mortality, while also streamlining the follow‐up process.

## 6 Conclusion

In conclusion, our study demonstrated that assisted home HD is non‐inferior to in‐center hemodialysis in terms of mortality. It also highlighted the detrimental impact of immobility on survival in the assisted home HD patients. Early identification of immobility and targeted interventions may improve patient outcomes. Further research is needed to explore the complex factors influencing mortality and to refine care strategies that enhance both survival and quality of life for assisted home HD patients.

## Conflicts of Interest

The authors declare no conflicts of interest.

## Supporting information


**Data S1** Supporting Information.

**Data S2** Supporting Information.

**Data S3** Supporting Information.

**Data S4** Supporting Information.

## Data Availability

The data that support the findings of this study are available on request from the corresponding author. The data are not publicly available due to privacy or ethical restrictions.
