# Supplementary material for: Exploring Mortality and Associated Risks Among Assisted Home Hemodialysis Patients in Qatar
Source: Hemodial Int. 2025 Mar 28;29(4):487–95. doi: 10.1111/hdi.13236 (PMC12531942; doi:10.1111/hdi.13236)
Supplement: Supplementary file 2 — Data S2 Supporting Information. [file HDI-29-487-s001.docx]

**Title Page:**

**Exploring Mortality and Associated Risks Among Assisted Home Hemodialysis Patients in Qatar**

**Short Title: Assisted Home Hemodialysis and Mortality in Qatar**

Abdullah I. Hamad, MD^1^; Amani Z. Zidan, PhD^2^; Mostafa F. Elshirbeny, MD^1^; Fadwa S. Al-Ali, MD^1^; Tarek A. Ghonimi, MD^1^; Mohamed Y. Abdelhadi, MD^1^; Mossab Filali, MD^3^; Ahmed Awaisu, PhD^2^; Rania A. Ibrahim, RN^1^; Mohamad M. Alkadi, MD^1^; Hassan A. Al-Malki, MD^1^

^1^Division of Nephrology, Department of Medicine, Hamad Medical Corporation, Doha, Qatar.

^2^Department of Clinical Pharmacy and Practice, College of Pharmacy, QU Health, Qatar University, Doha, Qatar.

^3^Metcocare Company, Doha, Qatar.

**Correspondence:** Abdullah Ibrahim Hamad, Division of Nephrology, Hamad General Hospital, Doha, Qatar. Fax:44394805, Phone: 44394854, **E-mail:** [ahamad9@hamad.qa](mailto:ahamad9@hamad.qa), ORCID number: 0000-0003-4677-7686

This study Approved by the Institutional Review board (IRB) for the Medical Research Center at Hamad Medical Corporation (HMC), Doha, Qatar (MRC-01-23-781; 20 February 2023 and MRC-01-24-014; 13 June 2024).
