# Supplementary material for: Exploring Mortality and Associated Risks Among Assisted Home Hemodialysis Patients in Qatar
Source: Hemodial Int. 2025 Mar 28;29(4):487–95. doi: 10.1111/hdi.13236 (PMC12531942; doi:10.1111/hdi.13236)
Supplement: Supplementary file 3 — Data S3 Supporting Information. [file HDI-29-487-s003.pdf]

مؤسسة حمد الطبية
Hamad Medical Corporation
HEALTH • EDUCATION • RESEARCH صحة • تعليم • بحوث

**APPROVAL LETTER**
**MEDICAL RESEARCH CENTER**
**HMC, DOHA-QATAR**

**Date: 20 February 2023**

**Dr. Abdullah Ibrahim Hamad**
**Senior Consultant, Medicine**
**Hamad General Hospital (HGH)**
**Hamad Medical Corporation**
**Doha-Qatar**

| | |
|---|---|
| **Protocol No.** | MRC-01-23-081 |
| **Study Title** | Efficiency and Safety of Assisted Home Hemodialysis Program in the State of Qatar |
| The above titled research study has been approved to be conducted in HMC and is summarized below: | |
| **Study type** | Data Review |
| **Data Collection Period** | 01/07/2021 to 31/12/2022 |
| **Team Member List** | Dr. Abdullah Ibrahim Hamad, Dr. Mohamed Yahya A. Mohamed, Dr. Mostafa Fottoh Abdelmagid M. Elshirbeny |
| **Review Type** | 'Exempt' under MOPH guidelines<br>Category 3: Research involving the collection or study of existing: Data, documents, records and the information is recorded by the investigator in such a manner that subjects cannot be identified, directly or through identifiers linked to the subjects. |
| **Decision** | Approved |
| **Hospitals/ Facilities Approved** | Hamad General Hospital (HGH) |

This study must be conducted in full compliance with all the relevant sections of the Rules and Regulations for Research at HMC, and the Medical Research Center should be notified immediately of any proposed changes to the study protocol that may affect the 'exempt' status of this study. Wherever amendments to the initial protocol are deemed necessary, it is the responsibility of the Principal Investigator to ensure that appropriate reviews and renewed approvals are in place before the study will be allowed to proceed.

Please note that only **research documentation currently uploaded in ABHATH** is to be utilized at any stage in the conduct of this study. The research team must ensure that changes and progress on the study are appropriately recorded in ABHATH, the online research system of the Medical Research Center. The PI must ensure that any link to patient identifiers is destroyed after data collection and data security is maintained.

We wish you success in this research and await the outcomes in due course.

Yours Sincerely,

Prof. Michael Paul Frenneaux
Chief of Scientific, Academic and Faculty Affairs
Hamad Medical Corporation

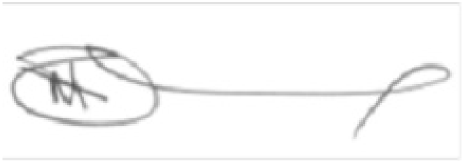

Date: 20 February 2023
