# Supplementary material for: Exploring Mortality and Associated Risks Among Assisted Home Hemodialysis Patients in Qatar
Source: Hemodial Int. 2025 Mar 28;29(4):487–95. doi: 10.1111/hdi.13236 (PMC12531942; doi:10.1111/hdi.13236)
Supplement: Supplementary file 1 — Data S1 Supporting Information. [file HDI-29-487-s002.pdf]

Cover Letter

Dear Editor-in-Chief:

We are submitting our original article for publication in Hemodialysis International entitled "**Exploring Mortality and Associated Risks Among Assisted Home Hemodialysis Patients in Qatar**".

This paper is authored by Abdullah I. Hamad, MD[1]; Amani Z. Zidan, PhD[2]; Mostafa F. Elshirbeny, MD[1]; Fadwa S. Al-Ali, MD[1]; Tarek A. Ghonimi, MD[1]; Mohamed Y. Abdelhai, MD[1]; Mossab Filali, MD[3]; Ahmed Awaisu, PhD[2], Rania A. Ibrahim, RN[1]; Mohamad M. Alkadi, MD[1]; Hassan A. Al-Malki, MD[1].

[1]Division of Nephrology, Department of Medicine, Hamad Medical Corporation, Doha, Qatar.

[2]Department of Clinical Pharmacy and Practice, College of Pharmacy, QU Health, Qatar University, Doha, Qatar.

[3]Metcocare Company, Doha, Qatar.

This article aimed to analyze all-cause mortality among patients undergoing AHHD in Qatar and to identify key contributing factors. Secondarily, it focused on examining patient characteristics, demographics, and comorbidities. This manuscript will interest your readers, and we hope to consider it for publication in your esteemed journal. All listed authors have contributed significantly, and all authors agree with the manuscript's content.

All authors have declared that no competing interests exist. The authors received no specific funding for this work. All listed authors confirm that neither the manuscript nor any parts of its content are currently under consideration or published in another journal, and they have approved the manuscript and agree with its submission to https://onlinelibrary.wiley.com/journal/15424758.

We successfully had a prior publication with Hemodialysis International

- Hemodial Int. 2016 Apr;20(2):204-7. PMID: 26501237. DOI: 10.1111/hdi.12379
- Randomized Controlled Trial, Hemodial Int. 2015 Jan; PMID: 24894344, DOI: 10.1111/hdi.12181

Thank you for your consideration. We look forward to hearing from you,

Corresponding author:

Abdullah Hamad, MD
Email: ahamad9@hamad.qa
